# ProFITS of maize: a database of protein families involved in the transduction of signalling in the maize genome

**DOI:** 10.1186/1471-2164-11-580

**Published:** 2010-10-19

**Authors:** Yi Ling, Zhou Du, Zhenhai Zhang, Zhen Su

**Affiliations:** 1 State Key Laboratory of Plant Physiology and Biochemistry, College of Biological Sciences, China Agricultural University, Beijing, 100193, PR China

## Abstract

**Background:**

Maize (*Zea mays *ssp. *mays *L.) is an important model for plant basic and applied research. In 2009, the B73 maize genome sequencing made a great step forward, using clone by clone strategy; however, functional annotation and gene classification of the maize genome are still limited. Thus, a well-annotated datasets and informative database will be important for further research discoveries. Signal transduction is a fundamental biological process in living cells, and many protein families participate in this process in sensing, amplifying and responding to various extracellular or internal stimuli. Therefore, it is a good starting point to integrate information on the maize functional genes involved in signal transduction.

**Results:**

Here we introduce a comprehensive database 'ProFITS' (Protein Families Involved in the Transduction of Signalling), which endeavours to identify and classify protein kinases/phosphatases, transcription factors and ubiquitin-proteasome-system related genes in the B73 maize genome. Users can explore gene models, corresponding transcripts and FLcDNAs using the three abovementioned protein hierarchical categories, and visualize them using an AJAX-based genome browser (JBrowse) or Generic Genome Browser (GBrowse). Functional annotations such as GO annotation, protein signatures, protein best-hits in the *Arabidopsis *and rice genome are provided. In addition, pre-calculated transcription factor binding sites of each gene are generated and mutant information is incorporated into ProFITS. In short, ProFITS provides a user-friendly web interface for studies in signal transduction process in maize.

**Conclusion:**

ProFITS, which utilizes both the B73 maize genome and full length cDNA (FLcDNA) datasets, provides users a comprehensive platform of maize annotation with specific focus on the categorization of families involved in the signal transduction process. ProFITS is designed as a user-friendly web interface and it is valuable for experimental researchers. It is freely available now to all users at http://bioinfo.cau.edu.cn/ProFITS.

## Background

Maize (*Zea mays *ssp. *mays *L.) is an important economic crop, and has served as a model organism for plant genetic research for several decades. The B73 maize genome was sequenced in 2009 [1-3], providing unprecedented opportunities for genome-wide annotation, classification and comparative genomics research. However, the comprehensive maize genome sequence repositories, MaizeSequence http://www.maizesequence.org[1] and maizeGDB http://www.maizegdb.org/[4] provide limited information concerning gene families' categorization. The thriving of research discoveries may be hampered under these circumstances.

Signal transduction is a fundamental biological process in living cells for sensing, amplifying and responding to various extracellular or internal stimuli [5]. Many gene products (proteins) are involved in this process. During the signal transduction process, the status of protein-protein interaction, protein three-dimensional architecture, and the localization of proteins could be altered by rapid changes in protein activities or stabilities. Protein phosphorylation and ubiquitination are two major donators of these changes through post-translation covalent modification. Furthermore, when they are associated with transcription factors (TFs) that can lead to the multitude transcription cascades, these proteins act as switches allowing the proper and timely response of signal information flow and avoiding overreaction. In the past two decades, identifying the components involved in signal transduction and determining specific signalling pathways have both been functional research hotspots. However, genome-wide classification of gene families involved in signal transduction of maize is still limited.

With the aim to facilitate studies on signal transduction in the maize genome, we developed the 'ProFITS' (Protein Families Involved in the Transduction of Signalling) of maize, a database which categorizes TFs, protein kinases/phosphatases (PKs/PPs) and ubiquitin-proteasome-system (UPS)-related genes in maize.

## Construction and content

### Data acquisition

The B73 maize genome dataset (version 4a.53) which includes gene, transcript and protein sequences were downloaded from MaizeSequence http://www.maizesequence.org/index.html[1]. Four maize full-length cDNA (FLcDNA) datasets [3,6-8] were obtained from GenBank [9] by the searching key 'FLI-CDNA'. To the FLcDNA dataset generated by Alexandrov [8], only high quality sequences labelled as 'completed cds' were selected for further analysis. To those FLcDNAs whose corresponding protein sequences were not available in GenBank, the EMBOSS suite [10] was applied for protein translation and the longest one of each FLcDNA was selected for further analysis. In addition, consensus sequences of TF binding sites (TFBS) were retrieved from two publicly accessible comprehensive plant *cis*-element databases, PLACE [11] and AtcisDB [12]. These two datasets were further merged into one by performing manual curation that low-quality or redundant TFBS consensus sequences were filtered or integrated. Furthermore, mutant information including mutant gene name, phenotype and location were obtained from MaizeGDB [4].

### Comprehensive annotation to the maize genome and FLcDNA sequences

First of all, InterProScan[13] was performed against the maize genome protein sequences and FLcDNA translations, and GO (gene ontology) [5] annotations were generated based on InterProScan results. In addition to InterProScan, Pfam[14] search was implemented separately using the newest version of Pfam database (Version 24.0, as of July 2010), because Pfam accessions were key identifiers used for TF classification. The gathering cut-off (-cut_ga), which is the minimum score a sequence must attain when building a full alignment of a Pfam entry, is applied as threshold. After that, the FLcDNA sequences were localized to the maize genome using GMAP [15] and correlated with maize genome transcripts using BLAST search [16]. Appearance of TFBS within 3 kb upstream sequences of each transcript was also computed by short sequence match with curated binding site consensus sequences using regular expression method. Then, putative homologs in *Arabidopsis *and rice genomes were identified using BLAST (*E*-value ≤ 1e-40 and Coverage ≥ 0.5).

**Figure 1:**
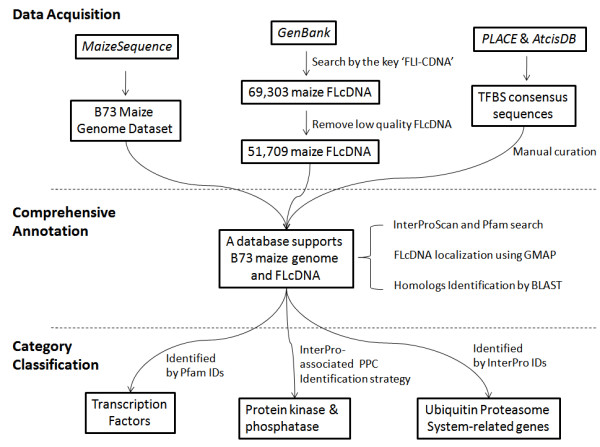
**Flowchart of data acquisition, comprehensive annotation and category classification in ProFITS**.

After series analyses above of the maize genome and FLcDNA data, we integrated the comprehensive annotation into ProFITS (see flowchart, Figure 1). All the data were made easily accessible and searchable.

### Category classification

We specifically classified three protein families involved in signal transduction: the TFs, the PKs/PPs and the UPS-related genes. Different strategies were designed and depicted as follow.

The identification approach of TFs is adopted from PlnTFDB [17], that TFs were predicted and classified based on protein domains identified by the Pfam search. For each TF family, there exists one or more required domains, while several families contain forbidden domains (See detailed rules in Additional File 1).

As for PKs/PPs, a modified PlantsP kinase Classification/PlantsP Phosphatase Classification (PPC) [18] is used for family classification. The H and No_PPC (not included in PPC yet) classes were added in this modified PPC system. The H class consists of two-component system related genes (e.g. histidine kinases), while No_PPC contains Hpt genes, casein kinase II and other kinases/phosphatases that cannot be classified in the original PPC classification. The sequences associated with required protein domains defined by InterPro accessions (which generated by InterProScan) were selected firstly. Then BLAST (*E*-value ≤ 1e-10 and Coverage ≥ 0.5) was done on candidate sequences against PPC classified Arabidopsis PKs/PPs sequences. The candidates were assigned to different PPC groups according to their best hit in the reference. The required InterPro accessions and a modified PPC criterion which intend to gather all the protein phosphorylation related genes in one category can be explored in Additional File 1.

Lastly, we identified UPS-related genes employing same method as in plantsUPS [19]. A group of InterPro accessions (see Additional File 1) were used for classification of different UPS-related gene families. Since there is no consensus accessions for RBX (Ring-Box) and DDB which is a component of CDD (CUL4-RBX1-CDD complex) families, BLAST search (*E*-value ≤ 1e-10 and Coverage ≥ 0.5) against protein sequences of these family members in *Arabidopsis *were implemented for identification.

### Database architecture

We constructed and configured ProFITS upon a typical LAMP (Linux + Apache + MySQL + PHP) platform. The dataset was stored in MySQL 5.0 http://www.mysql.com, and the web interface was built by PHP scripts http://www.php.net on Red Hat Linux, powered by an Apache server http://www.apache.org. Server-side scripts were developed using Python http://www.python.org.

## Utility and results

### Web interface overview

**Figure 2:**
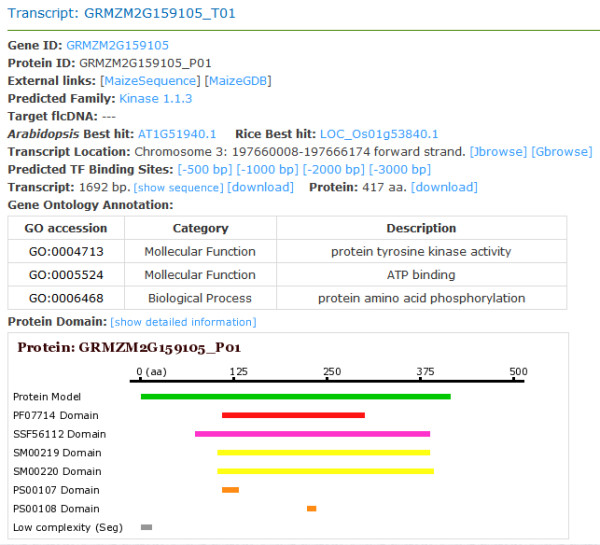
**Snapshot of information page of transcript 'GRMZM2G159105_T01' **. Detailed annotations are provided in the page, and linkages to other functional annotations are available.

In ProFITS, the TFs are all displayed in flat HTML tables, PKs/PPs and UPS-related genes are represented in a hierarchical tree mode. When exploring a particular family in these three categories, the genes including their transcripts and FLcDNAs in the family are simultaneously accessible, including the BLAST best-hits in *Arabidopsis*. The genome dataset is displayed on two levels (gene and transcript levels) as one gene may have one or more corresponding transcripts. In the page for gene level, comprehensive annotations (e.g. gene sequences, corresponding transcripts and mutant information) are provided. In the page for transcript level, more information generated by protein signatures analysis and gene function prediction is displayed because protein sequence is available for each transcript (Figure 2). Moreover, besides basic information similar to that of the gene-level page, users can check GO annotation, protein best-hits in the *Arabidopsis *and rice genomes, protein signatures and pre-calculated TFBS information in the transcript-level page. More detailed information containing TFBS consensus, promoter sequence and related annotations are available through links in the transcript-level pages. The FLcDNA annotation pages provide similar content as transcript-level pages.

### Feature tools and functionalities

ProFITS provides several analysis and exploration tools to facilitate users' research. An advanced search tool in ProFITS supports not only maize sequence IDs, but also IDs of *Arabidopsis *or rice, and *Arabidopsis *gene names. Additionally, we integrated an adopted GO enrichment analysis tool from agriGO [20], which facilitates users to uncover hidden biological meanings from a user-prepared list of gene IDs.

Genome browsers have been shown as one kind of useful tools in inspecting sequence structures and locations in a direct and visualized way - thus we set up and configured two different browsers, GBrowse [21] and JBrowse (Additional File 2) [22], catering to users' different requirements. Mutual links between the database and GBrowse/JBrowse are available so that users can easily switch aspects of the investigation to interesting targets.

### Statistics of three identified categories in ProFITS

In ProFITS, there are 32,540 genes and 53,764 transcripts of the maize genome [1], and 51,709 FLcDNA sequences. There were 2,505 genes identified as TFs in the maize genome, distributed in 80 different TF families; and 1,046 genes were identified as PKs/PPs. Lastly, 1,044 genes were characterized in the 12 UPS-related gene families (see statistical summary of three categories of the maize genome in Table 1).

**Table 1:** Total number of three identified categories in ProFITS

	Transcription Factor	Protein kinase/phosphatase	UPS-involved proteins
**Gene Model**	2505	1046	1044
**Transcript**	3509	1510	1585
**Full length cDNA**	2801	1031	1170

## Discussion

Although information concerning maize TFs and UPS-related genes can be found in PlnTFDB [17] and PlantsUPS [19], a complete profile of these two categories in the maize genome is still deficient. Based on gene annotation of the B73 maize genome (version 4a.53) and FLcDNA datasets, ProFITS provides a basic platform for maize functional genome research - the three key categories involved in signal transduction are particularly identified and classified. In addition, the predicted TFBS of genes together with TFs in ProFITS may provide clues to determine the possible effective TFs in a specific signal transduction pathway.

**Figure 3:**
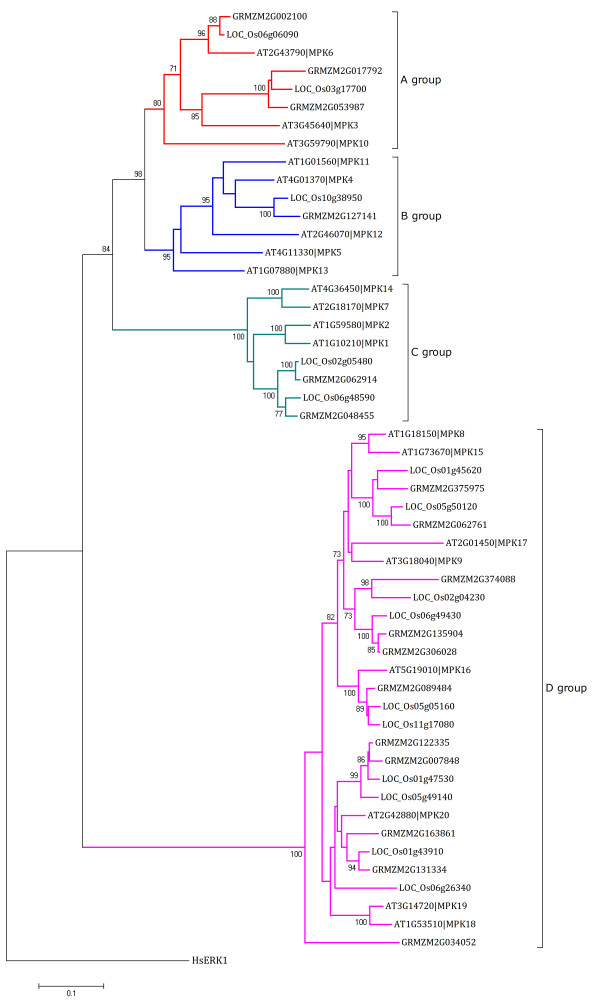
**Phylogenetic tree of *Arabidopsis*, rice and maize MAPK genes **. There are 20, 15, 17 MAPK genes from *Arabidopsis*, rice and maize, respectively, all of which contain an activation loop -TXY- motif. One MAPK gene from human (HsERK1) was used as an outgroup. The protein kinase domains of each sequence were predicted by Pfam and aligned by ClustalW [27] using the parameters: Pairwise alignment - Gap opening, 35.0, Gap extension, 0.75; Multiple alignment - Gap opening, 15.0, Gap extension, 0.30. A Neighbour-Joining bootstrapped tree was constructed by MEGA 4.0 [28] using pairwise deletion and JTT matrix-based model, and adjusted manually.

Completed profiles of *Arabidopsis *and rice PKs/PPs can be found in PlantsP [18] and RKD [23]; however, a similar categorization is limited in maize. In ProFITS, we identified 1,046 PK/PP genes and classified them using an InterProScan-associated PPC system. Compared with *Arabidopsis *and rice (1,168 and 1,467 genes, respectively) [18,23], the total number of maize PK/PP genes is relatively small. This may due to our more stringent identification method of applying InterPro accessions in pre-selection. We chose Mitogen Activated Protein Kinase (MAPK) subfamily from *Arabidopsis*, rice and maize for phylogenetic tree analysis using similar parameters as Hamel et. al. [24] (see Figure 3). Four clades were detected in the phylogenetic tree which is same as previous report [24]. Interestingly, MAPK members from rice and maize, both of which are monocot, are tend to be clustered on the same branches.

Jasmonate (JA) is a plant hormone (phytohormone) which participates in multiple developmental processes. The core of the JA-signalling module in *Arabidopsis*, SCF^COI1^/JAZ/MYC2, has been defined [25]. SCF^COI1 ^is an E3 ubiquitin ligase complex. After hormone perception by SCF^COI1^, JAZ (JAsmonate ZIM domain) repressors are targeted for proteasome degradation, releasing MYC2 and de-repressing transcriptional activation [26]. We checked the putative maize homologs of these genes using reciprocal BLAST (data not shown), and found that they were all in the corresponding categories of ProFITS.

We collected all 1,230 *Arabidopsis *genes classified in the signal transduction process (GO:0007165), and then explored their annotation of molecular function. Interestingly, among 1,169 genes annotated to have catalytic activities, > 60% have protein kinase activity (725) and about 10% have phosphatase activity (133). Only 0.8% of genes have protein ligase activity; however, this is threefold that of the 0.28% of all annotated with protein ligase activity genes in the *Arabidopsis *genome, which indicates their important roles in signal transduction processes. Other genes such as receptors, TFs, two-component response regulators and protein phosphatase type 2A regulators are under molecular transducer activity, transcription regulator activity and enzyme regulator activity terms, respectively (see Additional File 3). The GO distribution is consistent with our definition of ProFITS.

As ProFITS provides a platform of maize information, its expansibility will be useful when new data is available or a new gene family needs to be categorized.

## Conclusions

ProFITS provides users with a comprehensive profile of genes involved in signal transduction. Sequences of the maize genome and four maize FLcDNA projects are available, making it valuable for experimental researchers. It is freely available now to all users at http://bioinfo.cau.edu.cn/ProFITS.

## Authors' contributions

YL performed protein kinases/phosphatases classification, and compiled the Background and Discussion parts of the manuscript. ZD performed data collection and annotation, the database and web server construction, and compiled the Results part of the manuscript. ZZ provided system support. ZS supervised the project. All authors read and approved the final manuscript.

## Supplementary Material

Additional file 1 **Classification rules in ProFITS**. Detailed classification rules including a modified PPC criterion which is intended to gather all the protein-phosphorylation-related genes into one category. Click here for file

Additional file 2 **Snapshot of JBrowse in ProFITS**. In ProFITS, the text annotation and graphical exploration are interrelated to each other. Click here for file

Additional file 3 **GO enrichment analysis on *Arabidopsis *genes**. The hierarchal GO graph of 1230 *Arabidopsis *genes involved in signal transduction are subjected to GO enrichment analysis using agriGO http://bioinfo.cau.edu.cn/agriGO/. The aspect of molecular function is presented here. Click here for file
