# Supplementary figures and images for: ProFITS of maize: a database of protein families involved in the transduction of signalling in the maize genome
Source: BMC Genomics. 2010 Oct 19;11:580. doi: 10.1186/1471-2164-11-580 (PMC3091727; doi:10.1186/1471-2164-11-580)

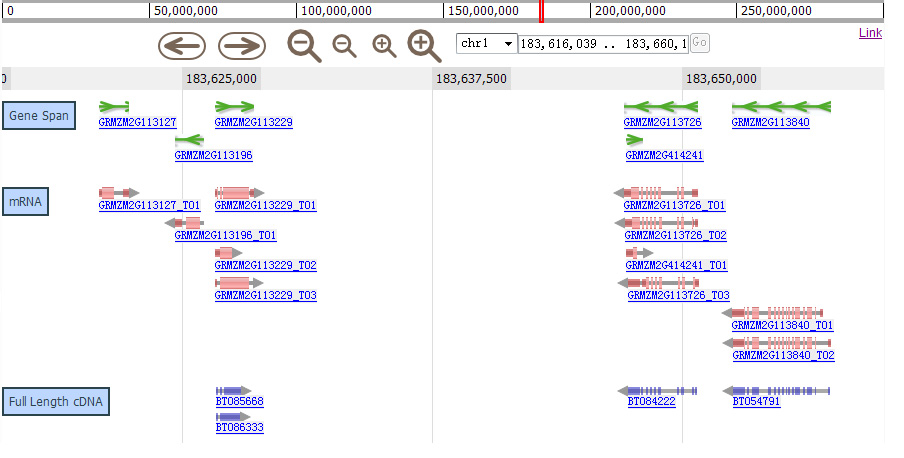

Supplement: Additional file 2 — Snapshot of JBrowse in ProFITS. In ProFITS, the text annotation and graphical exploration are interrelated to each other. [file 1471-2164-11-580-S2.JPEG]

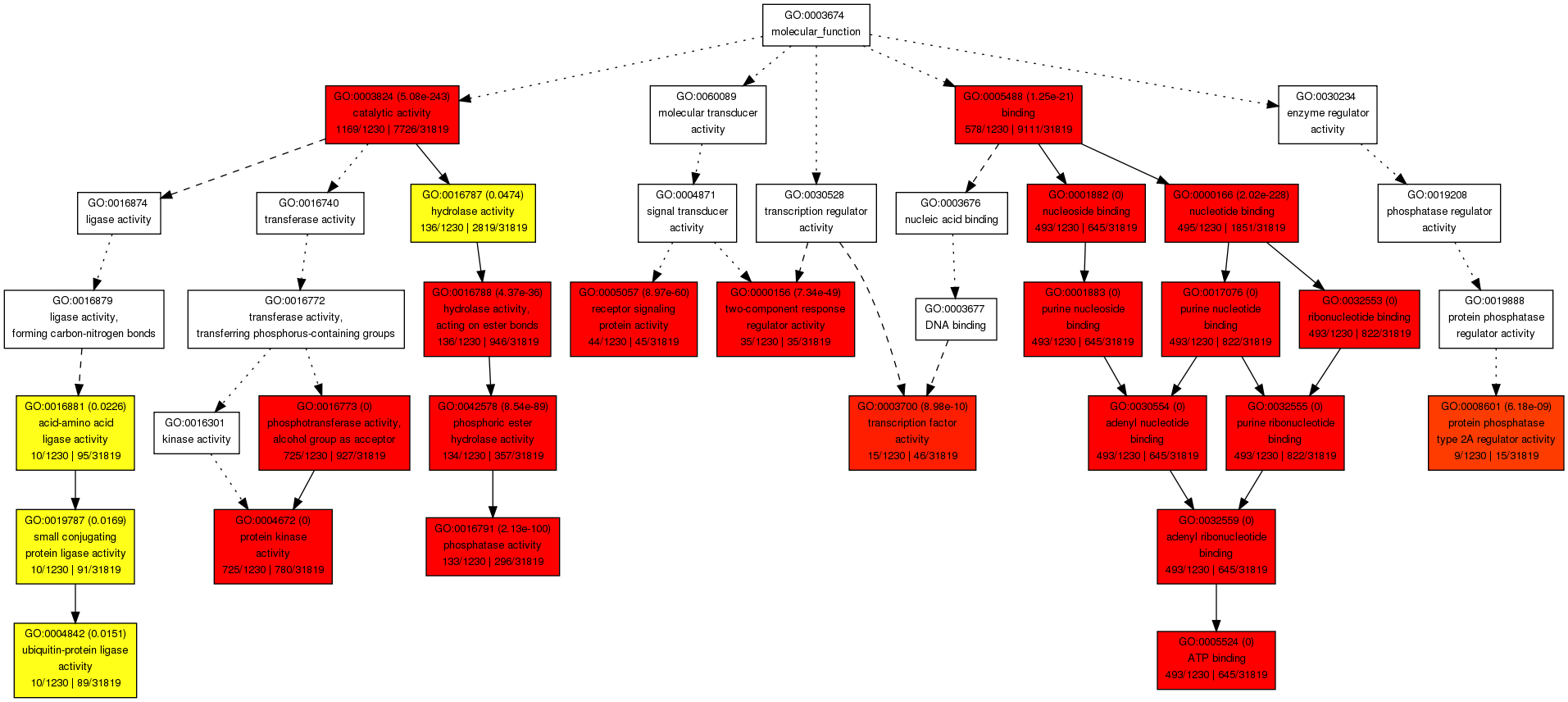

Supplement: Additional file 3 — GO enrichment analysis on Arabidopsis genes. The hierarchal GO graph of 1230 Arabidopsis genes involved in signal transduction are subjected to GO enrichment analysis using agriGO http://bioinfo.cau.edu.cn/agriGO/. The aspect of molecular function is presented here. [file 1471-2164-11-580-S3.JPEG]
